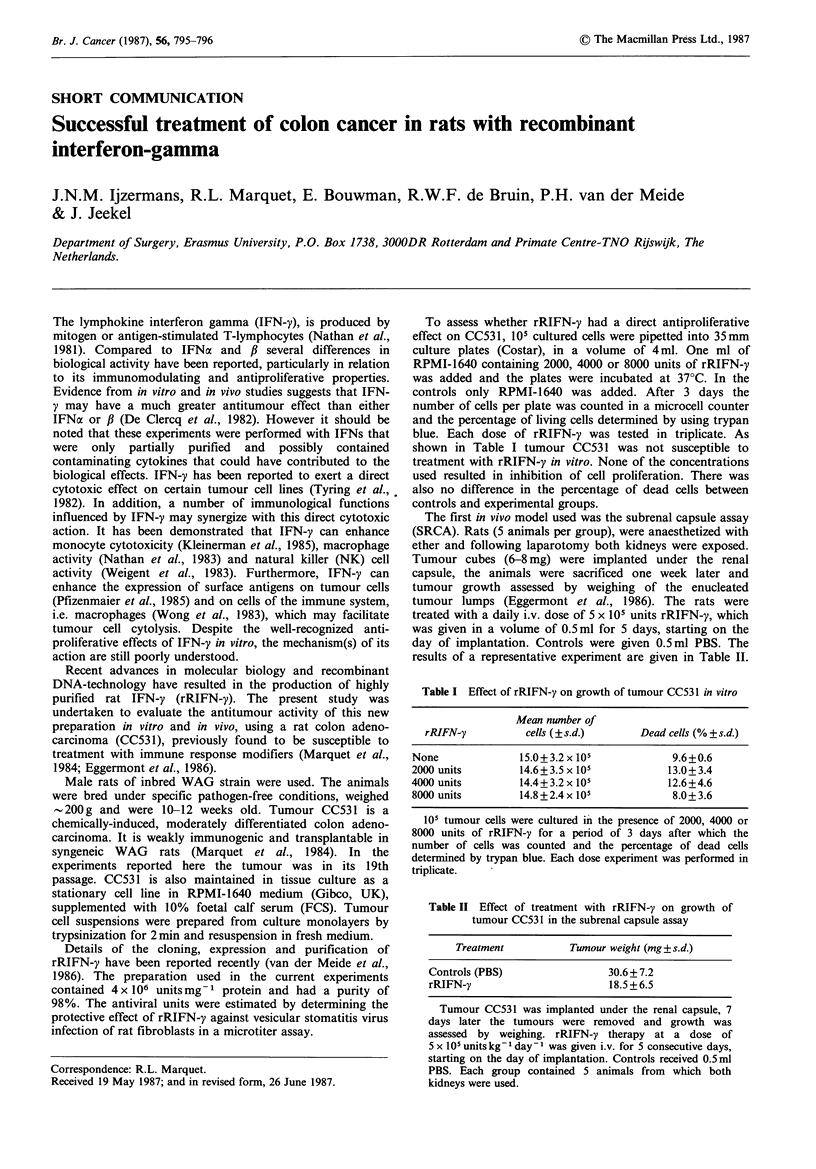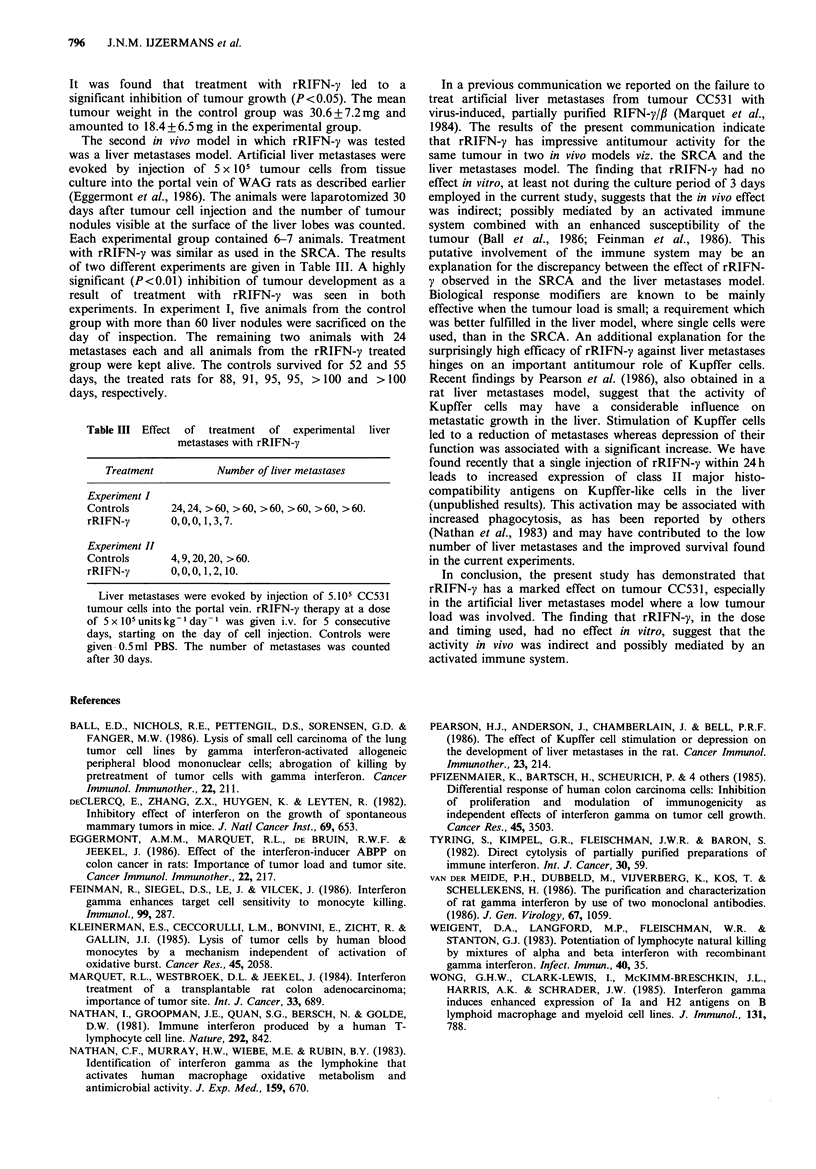# Successful treatment of colon cancer in rats with recombinant interferon-gamma.

**DOI:** 10.1038/bjc.1987.291

**Published:** 1987-12

**Authors:** J. N. Ijzermans, R. L. Marquet, E. Bouwman, R. W. de Bruin, P. H. van der Meide, J. Jeekel

**Affiliations:** Department of Surgery, Erasmus University, Rotterdam, The Netherlands.


Br. J. Cancer (1987), 56, 795–796

© The Macmillan Press Ltd., 1987

SHORT COMMUNICATION

### Successful treatment of colon cancer in rats with recombinant interferon-gamma

J.N.M. Ijzermans, R.L. Marquet, E. Bouwman, R.W.F. de Bruin, P.H. van der Meide & J. Jeekel

*Department of Surgery, Erasmus University, P.O. Box 1738, 3000DR Rotterdam and Primate Centre-TNO Rijswijk, The Netherlands.*

The lymphokine interferon gamma (IFN-γ), is produced by mitogen or antigen-stimulated T-lymphocytes (Nathan *et al.*, 1981). Compared to IFNα and β several differences in biological activity have been reported, particularly in relation to its immunomodulating and antiproliferative properties. Evidence from *in vitro* and *in vivo* studies suggests that IFN-γ may have a much greater antitumour effect than either IFNα or β (De Clercq *et al.*, 1982). However it should be noted that these experiments were performed with IFNs that were only partially purified and possibly contained contaminating cytokines that could have contributed to the biological effects. IFN-γ has been reported to exert a direct cytotoxic effect on certain tumour cell lines (Tyring *et al.*, 1982). In addition, a number of immunological functions influenced by IFN-γ may synergize with this direct cytotoxic action. It has been demonstrated that IFN-γ can enhance monocyte cytotoxicity (Kleinerman *et al.*, 1985), macrophage activity (Nathan *et al.*, 1983) and natural killer (NK) cell activity (Weigent *et al.*, 1983). Furthermore, IFN-γ can enhance the expression of surface antigens on tumour cells (Pfizenmaier *et al.*, 1985) and on cells of the immune system, i.e. macrophages (Wong *et al.*, 1983), which may facilitate tumour cell cytolysis. Despite the well-recognized anti-proliferative effects of IFN-γ *in vitro*, the mechanism(s) of its action are still poorly understood.

Recent advances in molecular biology and recombinant DNA-technology have resulted in the production of highly purified rat IFN-γ (rRIFN-γ). The present study was undertaken to evaluate the antitumour activity of this new preparation *in vitro* and *in vivo*, using a rat colon adenocarcinoma (CC531), previously found to be susceptible to treatment with immune response modifiers (Marquet *et al.*, 1984; Eggermont *et al.*, 1986).

Male rats of inbred WAG strain were used. The animals were bred under specific pathogen-free conditions, weighed ~200 g and were 10–12 weeks old. Tumour CC531 is a chemically-induced, moderately differentiated colon adenocarcinoma. It is weakly immunogenic and transplantable in syngeneic WAG rats (Marquet *et al.*, 1984). In the experiments reported here the tumour was in its 19th passage. CC531 is also maintained in tissue culture as a stationary cell line in RPMI-1640 medium (Gibco, UK), supplemented with 10% foetal calf serum (FCS). Tumour cell suspensions were prepared from culture monolayers by trypsinization for 2 min and resuspension in fresh medium.

Details of the cloning, expression and purification of rRIFN-γ have been reported recently (van der Meide *et al.*, 1986). The preparation used in the current experiments contained $4\times10^6$ units $mg^{-1}$ protein and had a purity of 98%. The antiviral units were estimated by determining the protective effect of rRIFN-γ against vesicular stomatitis virus infection of rat fibroblasts in a microtiter assay.

Correspondence: R.L. Marquet.

Received 19 May 1987; and in revised form, 26 June 1987.

To assess whether rRIFN-γ had a direct antiproliferative effect on CC531, $10^5$ cultured cells were pipetted into 35 mm culture plates (Costar), in a volume of 4 ml. One ml of RPMI-1640 containing 2000, 4000 or 8000 units of rRIFN-γ was added and the plates were incubated at 37°C. In the controls only RPMI-1640 was added. After 3 days the number of cells per plate was counted in a microcell counter and the percentage of living cells determined by using trypan blue. Each dose of rRIFN-γ was tested in triplicate. As shown in Table I tumour CC531 was not susceptible to treatment with rRIFN-γ *in vitro*. None of the concentrations used resulted in inhibition of cell proliferation. There was also no difference in the percentage of dead cells between controls and experimental groups.

The first *in vivo* model used was the subrenal capsule assay (SRCA). Rats (5 animals per group), were anaesthetized with ether and following laparotomy both kidneys were exposed. Tumour cubes (6–8 mg) were implanted under the renal capsule, the animals were sacrificed one week later and tumour growth assessed by weighing of the enucleated tumour lumps (Eggermont *et al.*, 1986). The rats were treated with a daily i.v. dose of $5\times10^5$ units rRIFN-γ, which was given in a volume of 0.5 ml for 5 days, starting on the day of implantation. Controls were given 0.5 ml PBS. The results of a representative experiment are given in Table II.

**Table I** Effect of rRIFN-γ on growth of tumour CC531 *in vitro*

| *rRIFN-γ* | *Mean number of cells (±s.d.)* | *Dead cells (% ±s.d.)* |
|---|---|---|
| None | $15.0\pm3.2\times10^5$ | $9.6\pm0.6$ |
| 2000 units | $14.6\pm3.5\times10^5$ | $13.0\pm3.4$ |
| 4000 units | $14.4\pm3.2\times10^5$ | $12.6\pm4.6$ |
| 8000 units | $14.8\pm2.4\times10^5$ | $8.0\pm3.6$ |

$10^5$ tumour cells were cultured in the presence of 2000, 4000 or 8000 units of rRIFN-γ for a period of 3 days after which the number of cells was counted and the percentage of dead cells determined by trypan blue. Each dose experiment was performed in triplicate.

**Table II** Effect of treatment with rRIFN-γ on growth of tumour CC531 in the subrenal capsule assay

| *Treatment* | *Tumour weight (mg±s.d.)* |
|---|---|
| Controls (PBS) | $30.6\pm7.2$ |
| rRIFN-γ | $18.5\pm6.5$ |

Tumour CC531 was implanted under the renal capsule, 7 days later the tumours were removed and growth was assessed by weighing. rRIFN-γ therapy at a dose of $5\times10^5$ units $kg^{-1}$ $day^{-1}$ was given i.v. for 5 consecutive days, starting on the day of implantation. Controls received 0.5 ml PBS. Each group contained 5 animals from which both kidneys were used.

It was found that treatment with rRIFN-γ led to a significant inhibition of tumour growth ($P<0.05$). The mean tumour weight in the control group was $30.6 \pm 7.2$ mg and amounted to $18.4 \pm 6.5$ mg in the experimental group.

The second *in vivo* model in which rRIFN-γ was tested was a liver metastases model. Artificial liver metastases were evoked by injection of $5 \times 10^5$ tumour cells from tissue culture into the portal vein of WAG rats as described earlier (Eggermont *et al.*, 1986). The animals were laparotomized 30 days after tumour cell injection and the number of tumour nodules visible at the surface of the liver lobes was counted. Each experimental group contained 6–7 animals. Treatment with rRIFN-γ was similar as used in the SRCA. The results of two different experiments are given in Table III. A highly significant ($P<0.01$) inhibition of tumour development as a result of treatment with rRIFN-γ was seen in both experiments. In experiment I, five animals from the control group with more than 60 liver nodules were sacrificed on the day of inspection. The remaining two animals with 24 metastases each and all animals from the rRIFN-γ treated group were kept alive. The controls survived for 52 and 55 days, the treated rats for 88, 91, 95, 95, >100 and >100 days, respectively.

**Table III** Effect of treatment of experimental liver metastases with rRIFN-γ

| *Treatment* | *Number of liver metastases* |
|---|---|
| *Experiment I* | |
| Controls | 24, 24, >60, >60, >60, >60, >60, >60. |
| rRIFN-γ | 0, 0, 0, 1, 3, 7. |
| *Experiment II* | |
| Controls | 4, 9, 20, 20, >60. |
| rRIFN-γ | 0, 0, 0, 1, 2, 10. |

Liver metastases were evoked by injection of $5.10^5$ CC531 tumour cells into the portal vein. rRIFN-γ therapy at a dose of $5 \times 10^5$ units kg$^{-1}$ day$^{-1}$ was given i.v. for 5 consecutive days, starting on the day of cell injection. Controls were given 0.5 ml PBS. The number of metastases was counted after 30 days.

In a previous communication we reported on the failure to treat artificial liver metastases from tumour CC531 with virus-induced, partially purified RIFN-γ/β (Marquet *et al.*, 1984). The results of the present communication indicate that rRIFN-γ has impressive antitumour activity for the same tumour in two *in vivo* models *viz.* the SRCA and the liver metastases model. The finding that rRIFN-γ had no effect *in vitro*, at least not during the culture period of 3 days employed in the current study, suggests that the *in vivo* effect was indirect; possibly mediated by an activated immune system combined with an enhanced susceptibility of the tumour (Ball *et al.*, 1986; Feinman *et al.*, 1986). This putative involvement of the immune system may be an explanation for the discrepancy between the effect of rRIFN-γ observed in the SRCA and the liver metastases model. Biological response modifiers are known to be mainly effective when the tumour load is small; a requirement which was better fulfilled in the liver model, where single cells were used, than in the SRCA. An additional explanation for the surprisingly high efficacy of rRIFN-γ against liver metastases hinges on an important antitumour role of Kupffer cells. Recent findings by Pearson *et al.* (1986), also obtained in a rat liver metastases model, suggest that the activity of Kupffer cells may have a considerable influence on metastatic growth in the liver. Stimulation of Kupffer cells led to a reduction of metastases whereas depression of their function was associated with a significant increase. We have found recently that a single injection of rRIFN-γ within 24 h leads to increased expression of class II major histocompatibility antigens on Kupffer-like cells in the liver (unpublished results). This activation may be associated with increased phagocytosis, as has been reported by others (Nathan *et al.*, 1983) and may have contributed to the low number of liver metastases and the improved survival found in the current experiments.

In conclusion, the present study has demonstrated that rRIFN-γ has a marked effect on tumour CC531, especially in the artificial liver metastases model where a low tumour load was involved. The finding that rRIFN-γ, in the dose and timing used, had no effect *in vitro*, suggest that the activity *in vivo* was indirect and possibly mediated by an activated immune system.

### References

BALL, E.D., NICHOLS, R.E., PETTENGIL, D.S., SORENSEN, G.D. & FANGER, M.W. (1986). Lysis of small cell carcinoma of the lung tumor cell lines by gamma interferon-activated allogeneic peripheral blood mononuclear cells; abrogation of killing by pretreatment of tumor cells with gamma interferon. *Cancer Immunol. Immunother.*, **22**, 211.

DECLERCQ, E., ZHANG, Z.X., HUYGEN, K. & LEYTEN, R. (1982). Inhibitory effect of interferon on the growth of spontaneous mammary tumors in mice. *J. Natl Cancer Inst.*, **69**, 653.

EGGERMONT, A.M.M., MARQUET, R.L., DE BRUIN, R.W.F. & JEEKEL, J. (1986). Effect of the interferon-inducer ABPP on colon cancer in rats: Importance of tumor load and tumor site. *Cancer Immunol. Immunother.*, **22**, 217.

FEINMAN, R., SIEGEL, D.S., LE, J. & VILCEK, J. (1986). Interferon gamma enhances target cell sensitivity to monocyte killing. *Immunol.*, **99**, 287.

KLEINERMAN, E.S., CECCORULLI, L.M., BONVINI, E., ZICHT, R. & GALLIN, J.I. (1985). Lysis of tumor cells by human blood monocytes by a mechanism independent of activation of oxidative burst. *Cancer Res.*, **45**, 2058.

MARQUET, R.L., WESTBROEK, D.L. & JEEKEL, J. (1984). Interferon treatment of a transplantable rat colon adenocarcinoma; importance of tumor site. *Int. J. Cancer*, **33**, 689.

NATHAN, I., GROOPMAN, J.E., QUAN, S.G., BERSCH, N. & GOLDE, D.W. (1981). Immune interferon produced by a human T-lymphocyte cell line. *Nature*, **292**, 842.

NATHAN, C.F., MURRAY, H.W., WIEBE, M.E. & RUBIN, B.Y. (1983). Identification of interferon gamma as the lymphokine that activates human macrophage oxidative metabolism and antimicrobial activity. *J. Exp. Med.*, **159**, 670.

PEARSON, H.J., ANDERSON, J., CHAMBERLAIN, J. & BELL, P.R.F. (1986). The effect of Kupffer cell stimulation or depression on the development of liver metastases in the rat. *Cancer Immunol. Immunother.*, **23**, 214.

PFIZENMAIER, K., BARTSCH, H., SCHEURICH, P. & 4 others (1985). Differential response of human colon carcinoma cells: Inhibition of proliferation and modulation of immunogenicity as independent effects of interferon gamma on tumor cell growth. *Cancer Res.*, **45**, 3503.

TYRING, S., KIMPEL, G.R., FLEISCHMAN, J.W.R. & BARON, S. (1982). Direct cytolysis of partially purified preparations of immune interferon. *Int. J. Cancer*, **30**, 59.

VAN DER MEIDE, P.H., DUBBELD, M., VIJVERBERG, K., KOS, T. & SCHELLEKENS, H. (1986). The purification and characterization of rat gamma interferon by use of two monoclonal antibodies. (1986). *J. Gen. Virology*, **67**, 1059.

WEIGENT, D.A., LANGFORD, M.P., FLEISCHMAN, W.R. & STANTON, G.J. (1983). Potentiation of lymphocyte natural killing by mixtures of alpha and beta interferon with recombinant gamma interferon. *Infect. Immun.*, **40**, 35.

WONG, G.H.W., CLARK-LEWIS, I., MCKIMM-BRESCHKIN, J.L., HARRIS, A.K. & SCHRADER, J.W. (1985). Interferon gamma induces enhanced expression of Ia and H2 antigens on B lymphoid macrophage and myeloid cell lines. *J. Immunol.*, **131**, 788.